# Multicountry Analysis of Spectrum of Clinical Manifestations of Children <5 Years of Age Hospitalized with Diarrhea

**DOI:** 10.3201/eid2512.180712

**Published:** 2019-12

**Authors:** Jillian Murray, S. Yati Soenarto, Nenny S. Mulyani, Pushpa S. Wijesinghe, Evans M. Mpabalwani, Julia C. Simwaka, Belem Matapo, Jason M. Mwenda, Gayane Sahakyan, Svetlana Grigoryan, Artavazd Vanyan, Sergey Khactatryan, Jennifer Sanwogou, Lúcia Helena de Oliveira, Gloria Rey-Benito, Gagandeep Kang, Fatima Serhan, Jacqueline E. Tate, Negar Aliabadi, Adam L. Cohen, the Global Rotavirus Surveillance Network Clinical Presentation Group

**Affiliations:** World Health Organization, Geneva, Switzerland (J. Murray, F. Serhan, A.L. Cohen);; Universitas Gadjah Mada/Dr. Sardjito Hospital, Yogyakarta, Indonesia (S.Y. Soenarto, N.S. Mulyani);; World Health Organization, South-East Asia Regional Office, New Delhi, India (P.S. Wijesinghe);; University Teaching Hospital, Lusaka, Zambia (E.M. Mpabalwani, J.C. Simwaka);; World Health Organization Country Office, Lusaka (B. Matapo);; World Health Organization Regional Office for Africa, Brazzaville, Republic of the Congo (J.M. Mwenda);; National Immunization Program, Yerevan, Armenia (G. Sahakyan);; National Centre of Disease Control and Prevention, Yerevan (G. Sahakyan, S. Grigoryan, A. Vanyan);; Ministry of Health of Armenia, Yerevan (G. Sahakyan, S. Grigoryan, A. Vanyan, S. Khactatryan);; Immunization and Epidemiology of Vaccine-Preventable Diseases Program, Yerevan (S. Grigoryan);; Pan American Health Organization, Washington, DC, USA (J. Sanwogou, L.H. de Oliveira, G. Rey-Benito);; Christian Medical College, Vellore, India (G. Kang);; Centers for Disease Control and Prevention, Atlanta, Georgia, USA (J.E. Tate, N. Aliabadi)

**Keywords:** multicountry analysis, spectrum, clinical manifestations, children, child surveillance, hospitalizations, rotavirus, viruses, enteric infections, diarrhea, India, Indonesia, Zambia, Armenia, Bolivia, El Salvador, Paraguay

## Abstract

After introduction of rotavirus vaccine, other pathogens might become leading causes of hospitalizations for severe diarrhea among children <5 years of age. Our study in 33 hospitals in 7 countries found acute gastroenteritis accounted for most (84%) reported hospitalizations of children with diarrhea. Bloody and persistent diarrhea each accounted for <1%.

Diarrhea is a leading cause of illness and death in children <5 years of age globally. The most common cause is rotavirus. Persons infected with this virus typically have acute watery diarrhea and gastroenteritis (*1**–**4*). As rotavirus vaccines are increasingly incorporated into national immunization programs globally and the proportion of diarrhea caused by rotavirus decreases, other causes of pediatric diarrhea (such as *Shigella* spp.) are responsible for an increasing proportion of diarrhea, and these pathogens might have different clinical manifestations (*5**–**7*).

Because of this evolving etiology of diarrhea in children, it is necessary to clarify the spectrum of clinical manifestations of diarrhea to better inform interventions and surveillance systems, particularly in low- and middle-income countries (LMICs), where the burden of diarrhea is highest (*1**–**4*). We report a spectrum of clinical manifestations for diarrheal illness reported in hospitalized children <5 years of age in 7 countries.

## The Study

Data collection and reporting to the Global Rotavirus Surveillance Network occurs as part of routine public health surveillance in participating countries and does not require human subjects review. As part of a larger study on the etiology of pediatric diarrhea in LMICs, we conducted a retrospective review of ward admission logbooks and electronic databases from 33 hospitals with pediatric services that conduct sentinel surveillance for rotavirus. Hospitals were chosen for this analysis from the World Health Organization (WHO)–coordinated Global Rotavirus Surveillance Network and the Indian National Rotavirus Surveillance Network (*8**–**10*).

A convenience sample of countries was chosen by using the following inclusion criteria: countries had >1 sentinel hospital reporting data to one of the surveillance networks above, sentinel sites in the country had 12 consecutive months of available logbook data for each year included in the analysis, and individual sentinel sites in the country enrolled >100 diarrhea case-patients each year. Sites were not eligible for inclusion in the larger study, and therefore our convenience sample, if they had participated in the Global Enteric Multicenter Study (*11*). We used a convenience sample to ensure that sites selected were available and able to perform the retrospective logbook review.

Participating countries performed retrospective manual reviews of admissions logbooks. All countries used paper-based surveillance logbooks except countries in the Americas and Zambia, which have electronic databases. Data were abstracted by hospital data managers for total diarrhea admissions for children <5 years of age in at least 2–3 of the previous 5 years for the following mutually exclusive clinical surveillance categories: acute gastroenteritis (watery, nonbloody diarrhea); bloody diarrhea (dysentery); persistent (chronic, nonacute) diarrhea; and other, nonspecified diarrhea (*12*). The other, nonspecified diarrhea category contains admissions that did not meet criteria for acute watery, bloody, or persistent diarrhea or for which the logbook information was insufficient to classify the case into a specific category. Aggregate numbers of diarrheal admissions were tallied for each category by month and year.

For countries with prevaccine and postvaccine introduction data, we compared the proportions of admissions for each diarrheal category in the preintroduction and postintroduction periods by using the χ^2^ test. We used Stata version 12 (https://www.stata.com) for all analyses.

We included 7 countries that had 33 sentinel surveillance hospitals in this analysis. Acute gastroenteritis accounted for most (median 84%) of hospitalized case-patients with diarrhea overall. Bloody diarrhea accounted for a median <1%, and persistent diarrhea accounted for a median of 0% (Table 1). The proportion of diarrhea cases classified as acute gastroenteritis varied from 41% to 96%; acute gastroenteritis also accounted for most cases in each country except for El Salvador, where 59% of cases were categorized as other, nonspecified. Four countries provided data disaggregated by sentinel site (Table 2). The proportion of total diarrhea admissions due to acute gastroenteritis varied within sites from year to year and between sites within the same country.

**Table 1:** Characteristics of children <5 y of age hospitalized for diarrhea in selected countries from the WHO-Coordinated Global Rotavirus Sentinel Surveillance Network and the Indian National Rotavirus Surveillance Network, 2009–2016*

Vaccine status and country/WHO region	No. sites (no. years data)	No. diarrhea cases	Clinical manifestations of diarrhea			
			Acute watery	Bloody	Persistent	Other, nonspecified†
Overall	33‡ (23)	42,632	29,853	358	1,467	10,954
Before§	13 (8)	11,637	10,396	321	55	865
India/SEAR	7 (3)	5,261	4,940 (94)	196 (4)	20 (<1)	105 (2)
Indonesia/SEAR	5 (2)	1,995	1,695 (85)	110 (6)	35 (2)	155 (8)
Zambia/AFR	1 (3)	4,381	3,761 (86)	15 (<1)	0 (0)	605 (14)
Median proportion	NA	NA	86	4	<1	8
After§	21 (15)	30,995	19,457	37	1,412	10,089
Armenia/EUR	2 (3)	8,938	7,414 (83)	0 (0)	1,412 (16)	112 (1)
Bolivia/AMR	6 (3)	4,505	3,229 (72)	0 (0)	0 (0)	1,276 (28)
El Salvador/AMR	8 (3)	13,321	5,466 (41)	0 (0)	0 (0)	7,855 (59)
Paraguay/AMR	4 (3)	1,515	1,454 (96)	0 (0)	0 (0)	61 (4)
Zambia/AFR	1 (2)	2,716	1,894 (70)	37 (1)	0 (0)	785 (29)
Median proportion	NA	NA	72	0	0	28
Overall median proportion	NA	NA	84	<1	0	11

*Values are no. (%) unless otherwise indicated. AFR, African region; AMR, Americas region; EUR, European region; SEAR, Southeast Asian region; WHO, World Health Organization; NA, not applicable. †Other category contains admissions that did not meet criteria for acute gastroenteritis, bloody or persistent diarrhea, or the logbook information was insufficient to classify the case into a specific category. ‡Includes only 1 site for Zambia, which has data both preintroduction and postintroduction. §Excludes year of vaccine introduction for Armenia, Bolivia, El Salvador, Paraguay, and Zambia.

**Table 2:** Proportion of children <5 years of age hospitalized for diarrhea who also had acute gastroenteritis at sentinel sites, WHO-Coordinated Global Rotavirus Sentinel Surveillance Network, 2013–2015*

Country	Year of vaccine introduction	National rotavirus vaccine coverage, 2016, % (*13*)	Site	Acute gastroenteritis, %†			
				2013	2014	2015	Weighted mean
Bolivia	2008	99	1	82	65	72	74
			2	81	82	96	85
			3	76	65	100	78
			4	56	49	46	52
			5	93	99	88	95
			6	53	50	37	48
			Total	74	68	71	72
El Salvador	2006	92	1	28	49	57	40
			2	40	38	24	35
			3	48	41	41	44
			4	51	34	30	40
			5	59	46	75	61
			6	44	29	19	30
			7	22	6	NA	14
			8	62	54	54	57
			Total	45	35	43	41
Paraguay	2009	93	1	96	97	100	98
			2	100	84	NA	89
			3	78	100	100	94
			4	100	97	100	99
			Total	92	97	100	96
Indonesia	NA	NA	1	NA	98	95	97
			2	NA	95	91	93
			3	NA	97	99	98
			4	NA	61	70	66
			5	NA	68	58	63
			Total	NA	83	86	85

*NA, not available; WHO, World Health Organization. †These calculations include nonspecified diarrhea in the denominator. Thus, individual site years might have higher percentage acute gastroenteritis if this category is excluded.

The proportions of bloody and persistent diarrhea were similar across years in the same country, but there were some differences in bloody diarrhea proportions between sites in the same country. In Indonesia, 4 sites had average proportions of <10% bloody diarrhea, but 1 site had an average of 28% across both years. Overall, a median of 11% of diarrheal cases were categorized as other, nonspecified.

Zambia provided 3 years of data from before and 2 years of data from after rotavirus vaccine introduction (Table 1). The proportion of diarrhea caused by acute gastroenteritis decreased from 86% in the preintroduction era to 70% in the postintroduction era (p<0.01). There was a concomitant increase in other, nonspecified diarrhea cases, from 14% to 29%, over the same period (p<0.01).

## Conclusions

The most common clinical manifestations of children with cases of diarrhea were acute watery diarrhea and gastroenteritis in the LMICs analyzed; cases classified as bloody and persistent diarrhea cases were rare. However, the proportions of different clinical manifestations of pediatric diarrhea varied between sentinel sites within a country and between countries and regions. Differences between sites within the same country could be caused by different hospital-specific practices for describing the clinical manifestation of diarrheal disease, disease referral and healthcare use patterns, or relative uptake of rotavirus vaccine in countries that have introduced vaccine.

In the period before a country introduces rotavirus vaccine, one of the main objectives of rotavirus surveillance is to provide data on burden of disease and to describe rotavirus disease epidemiology (*12*). In the period after rotavirus vaccine introduction, one of the main objectives of surveillance is to assess the effect of vaccine. Although patient admissions for bloody and persistent diarrhea constitute a smaller proportion of pediatric case-patients with diarrhea, excluding these patients in pediatric diarrhea surveillance might overestimate the proportion of total diarrhea cases as being positive for rotavirus. Although WHO recommends surveillance for acute watery diarrhea to monitor rotavirus disease, the case definition of diarrheal cases enrolled needs to be expanded to fully capture the changing etiology of disease in the post–rotavirus vaccine era (*12*,*14*).

Our study and analysis have several limitations, many of which are caused by the retrospective nature of logbook reviews. First, the review was conducted as a convenience sample of 7 countries. Therefore, findings might not be generalizable to every country. There are regional variations in the case definitions based on the practices of clinicians at individual sentinel hospitals. Large proportions of diarrhea cases were also categorized as other, nonspecified, which might have been defined differently locally or over time; logbooks often have inadequate information to classify each case into one of the specified categories used for this analysis. In addition, age stratification <5 years of age for diarrhea hospitalizations was not available. Last, site-specific differences, such as disease classification practices and healthcare use patterns, and regional differences, such as rurality, socioeconomic status, and prevalence of malnutrition, might also play a role in intracountry differences, and data on these characteristics for the hospitals included in this analysis were not available.

Monitoring bloody and persistent pediatric diarrhea in addition to acute gastroenteritis is useful for fully understanding the burden and etiology of diarrhea in children, especially after introduction of rotavirus vaccine. Expanding the case definition recommended by WHO for pediatric diarrhea surveillance to include other types of diarrhea would facilitate more robust disease estimates and monitor the rollout and effect of these vaccines once they are introduced.
